# Vacuum-and-solvent-free fabrication of organic semiconductor layers for field-effect transistors

**DOI:** 10.1038/srep14547

**Published:** 2015-09-29

**Authors:** Toshinori Matsushima, Atula S. D. Sandanayaka, Yu Esaki, Chihaya Adachi

**Affiliations:** 1 Center for Organic Photonics and Electronics Research, Kyushu University, 744 Motooka, Nishi, Fukuoka 819-0395, Japan; 2 Japan Science and Technology Agency (JST), ERATO, Adachi Molecular Exciton Engineering Project, 744 Motooka, Nishi, Fukuoka 819-0395, Japan

## Abstract

We demonstrate that cold and hot isostatic pressing (CIP and HIP) is a novel, alternative method for organic semiconductor layer fabrication, where organic powder is compressed into a layer shape directly on a substrate with 200 MPa pressure. Spatial gaps between powder particles and the other particles, substrates, or electrodes are crushed after CIP and HIP, making it possible to operate organic field-effect transistors (OFETs) containing the compressed powder as the semiconductor. The CIP-compressed powder of 2,7-dioctyl[1]benzothieno[3,2-b][1]benzothiophene (C8-BTBT) had a hole mobility of (1.6 ± 0.4) × 10^–2^ cm^2^/Vs. HIP of C8-BTBT powder increased the hole mobility to an amorphous silicon-like value (0.22 ± 0.07 cm^2^/Vs) because of the growth of the C8-BTBT crystallites and the improved continuity between the powder particles. The vacuum and solution processes are not involved in our CIP and HIP techniques, offering a possibility of manufacturing OFETs at low cost.

Organic field-effect transistors (OFETs) containing semiconductor layers that are based on small molecules, oligomers and polymers may act as switches and/or signal-processing elements for practical applications ranging from drive circuits of active matrix displays^1^,^2^ to physical, biological, and medical sensors^3^,^4^,^5^,^6^,^7^, actuators^8^ and radio-frequency identification tags^9^,^10^. To fabricate the organic semiconductor layers, two different processes have been mainly employed: vacuum vapor deposition and solution processing. Vacuum vapor deposition is a costly process because of the rather long processing time, waste of most of the expensive organic materials, and difficulty of large-scale production. Although vacuum vapor deposition has been the mainstream process, recent studies have considered solution processing, such as spin-coating^11^,^12^, ink-jet printing^13^,^14^, and blade coating^15^ as ideal candidates for the organic semiconductor layer fabrication. Solution processing is thought to significantly reduce the manufacturing costs because solution processing overcomes the above problems. Very high carrier mobilities over 10 cm^2^/Vs have already been reported from solution-processed organic layers and single crystals^12^,^14^. However, organic materials must be dissolved in organic solvents for solution processing. It is preferable not to use organic solvents, particularly halogenated solvents, for industrial manufacturing.

There have been several attempts towards completely vacuum-and-solvent-free fabrication of the organic semiconductor layers. S. Nagamatsu *et al.* used a friction-transfer process to fabricate OFETs^16^. A pellet of regioregular poly(3-dodecylthiophene) (P3DDT) was squeezed on a substrate heated at 370 K and then drawn to a certain direction under a N_2_ atmosphere, forming a layer wherein the P3DDT molecules are aligned along the drawing direction. The OFET with the friction-transferred P3DDT layer had a hole mobility (*μ*_h_) of 7.4 × 10^–4 ^cm^2^/V s in the alignment direction. M. Shtein *et al.* introduced organic vapor jet printing for direct and efficient organic semiconductor layer deposition^17^. In this method, a hot inert carrier gas entered the apparatus and picked up the organic vapor. The gas mixture was then ejected through a nozzle. The collimated vapor jet from the nozzle impinged onto a cooled substrate, forming a pentacene pattern with *μ*_h_ = 0.2 cm^2^/V s. M. Treier *et al.* developed a solid-to-solid transfer process^18^. Polycrystalline powder of cyclopentadithiophene-benzothiadiazole (CDT-BTZ) was sprinkled on a substrate and then thermally annealed at a mild temperature of 90 °C for 20–70 h in a N_2_ atmosphere. They obtained *μ*_h _= 3.7 × 10^–5^ cm^2^/Vs from the OFET based on the annealed CDT-BTZ powder because of improved adhesion to the channel and electrodes through thermal annealing. A. Kim *et al.* demonstrated a template-assisted self-assembly process^19^. Crystalline powder of 2,7-didecylbenzothienobenzothiophene (C10-BTBT) was placed at entrances of trenches formed between a substrate and a patterned polydimethylsiloxane (PDMS) stamp. The trenches were filled with liquid-crystalline C10-BTBT by a capillary effect above its isotropic phase temperature (122 °C), forming C10-BTBT micropatterns after removing the PDMS stamp. Excellent transistor performance was obtained from the micropatterned C10-BTBT (*μ*_h _= 1.74 cm^2^/V s).

Cold isostatic pressing (CIP) has been widely used to compress and mold metal, ceramic, plastic, or composite powders into a certain form^20^,^21^,^22^. Recently, an improvement in the mechanical characteristics and a 2000 times increase in hole mobility were achieved using CIP treatment of vacuum-deposited metal-free phthalocyanine (H_2_PC) layers with a 200 MPa pressure because the spatial gaps were crushed between H_2_PC grains^23^,^24^. In this study, the CIP process was applied to compress organic powder directly on a Si substrate equipped with a SiO_2_ insulator and Au source-drain electrodes for the OFET fabrication. Here, we demonstrate that operation of this OFET containing the CIP-compressed powder as the semiconductor is possible. The CIP-compressed powder of 2,7-dioctyl[1]benzothieno[3,2-b][1]benzothiophene (C8-BTBT) had *μ*_h_ = (1.6 ± 0.4) × 10^–2^ cm^2^/Vs in the saturation regime. Additionally, hot isostatic pressing (HIP) was used to compress C8-BTBT powder for a further improvement in OFET performance, resulting in *μ*_h _= 0.22 ± 0.07 cm^2^/V s, which is the same order of magnitude as the mobility of amorphous silicon. Vacuum and solution processes are not involved in our CIP and HIP techniques, offering a possibility of manufacturing future OFETs at low cost.

## Results

### Compression of organic powder using CIP

**Figure 1:**
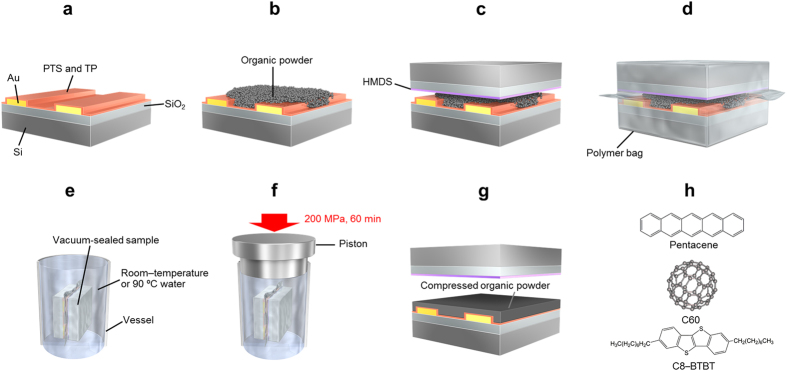
Illustrations of powder compression using CIP and HIP for OFET fabrication. (**a**) The SiO_2_ insulator was treated with PTS and the Au source-drain electrodes were treated with TP for the phenyl termination of the surfaces. (**b**) Purified degassed organic powder was put on the phenyl-terminated surfaces. (**c**) The powder was covered with another substrate treated with HMDS (methyl-terminated surface). (**d**) This sample was vacuum sealed inside a polymer bag. (**e**) The vacuum-sealed sample was immersed in water. The water was at room temperature for CIP and 90 °C for HIP. (**f**) A high pressure of 200 MPa was applied to the sample for 1 h through the water medium. (**g**) The compressed powder was formed on the phenyl-terminated substrate. (**h**) The chemical structures of pentacene, C60, and C8-BTBT used in this study. One of the authors (Toshinori Matsushima) drew the illustrations.

The powder compression process using CIP for the OFET fabrication is illustrated in Fig. 1. A SiO_2_ layer and Au source-drain electrodes formed on an *n*-type Si substrate were treated with phenyltrichlorosilane (PTS) and thiophenol (TP), respectively (Fig. 1a). The organic powder was placed on the treated substrate (Fig. 1b) and then covered by another Si substrate that was treated with hexamethyldisiloxane (HMDS) (Fig. 1c). This sandwich sample was transferred into a thin flexible polymer bag, which was then vacuum sealed (Fig. 1d). The bag was immersed in a metal vessel filled with room-temperature water (Fig. 1e). A pressure of 200 MPa was applied to the sample for 60 min through the water by pushing a metal piston into the vessel with an external pressurization source (Fig. 1f). After the CIP process, the bottom-contact bottom-gate FET containing the compressed powder as the semiconductor was obtained (Fig. 1g). Pentacene, carbon-60 (C60), or C8-BTBT were used as the organic semiconductors (Fig. 1h).

Surface treatments with PTS, TP, and HMDS are important for controlling the adhesion of the compressed powder to the substrate. Because both the organic powder and substrate surfaces treated with PTS and TP are chemically aromatic (through phenyl termination), the adhesion between organic powder and the phenyl-terminated surface is strong but the adhesion between organic powder and the non-aromatic methyl-terminated surface (the HMDS treated surface) is weak. Therefore, the compressed powder strongly adheres to the phenyl-terminated surface and easily peels away from the methyl-terminated surface. The compressed powder hardly delaminated from the phenyl-terminated substrate because of the strong adhesion even if the sample was strongly shaken.

**Figure 2:**
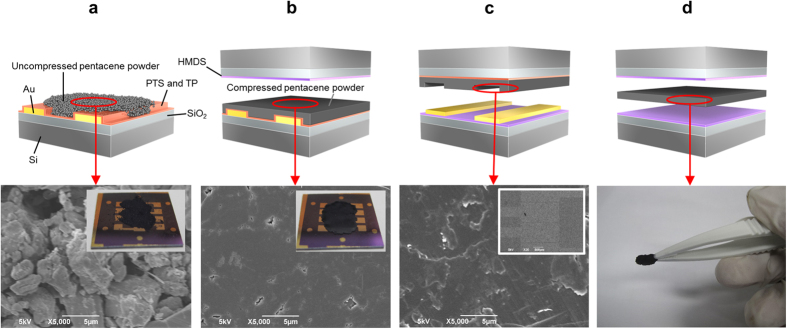
SEM images (×5,000 magnification) of (**a**) uncompressed pentacene powder, (**b**) CIP-compressed pentacene powder, and (**c**) channel region of the CIP-compressed pentacene powder and a photograph of (**d**) free-standing compressed pentacene powder. The insets in (**a**) and (**b**) are photographs of the uncompressed pentacene powder and CIP-compressed pentacene powder, respectively. The inset in (**c**) is the low-magnification SEM image (×30) of the channel region. The upper illustrations of each figure are the corresponding sample structures used to measure the SEM images and photographs. One of the authors (Toshinori Matsushima) drew the illustrations.

The photographs and scanning electron microscope (SEM) images of the uncompressed and CIP-compressed pentacene powders are shown in Fig. 2. A large number of the powder particles and spatial gaps between the powder particles were observed from the uncompressed powder (Fig. 2a). Therefore, the uncompressed powder had a matte surface with low light reflection (the inset of Fig. 2a). CIP at 200 MPa pressure enabled the individual particles to be less visible and the gaps to crush (Fig. 2b), resulting in a shiny surface of the compressed powder with high light reflection (the inset of Fig. 2b). The compressed powder looked like a film that was melted and then cooled. The bottom substrate with the source-drain electrodes was methyl terminated and the upper substrate was phenyl terminated. In this case, the compressed powder adheres to the upper phenyl-terminated substrate but peels away from the bottom methyl-terminated substrate with the source-drain electrodes. The channel region of this sample, where a current flow occurs under the FET operation, can be directly analyzed with SEM (Fig. 2c). The channel region was relatively flat and a terrace structure was observed in the channel region. The hollows deriving from the source-drain electrodes were confirmed in the low-magnification SEM image of the channel region (the inset of Fig. 2c). When the organic power was put between two methyl-terminated substrates and then CIP performed, the free-standing compressed powder was obtained because the compressed powder peeled away from both methyl-terminated substrates. The free-standing compressed powder was relatively hard and we could pick up the compressed powder using a pair of tweezers (Fig. 2d).

### Structural characteristics of CIP-compressed powders

**Figure 3:**
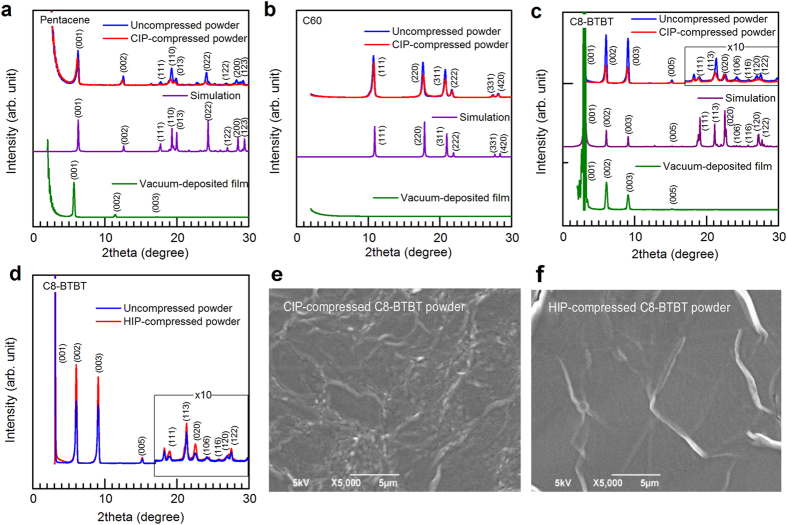
XRD profiles of the uncompressed and CIP-compressed powders and vacuum-deposited films of (**a**) pentacene, (**b**) C60, and (**c**) C8-BTBT and (**d**) the uncompressed and HIP-compressed C8-BTBT powders. Simulated XRD profiles are also shown in each figure. SEM images of the channel regions of (**e**) CIP- and (**f**) HIP-compressed C8-BTBT powders. A sample structure similar to Fig. 2c was used to measure the SEM images of the channel regions.

To investigate the structural characteristics, X-ray diffraction (XRD) profiles of the uncompressed and CIP-compressed powders of pentacene, C60, and C8-BTBT were measured (Fig. 3a–c). There were many diffraction peaks arising from several types of interlayer spacing in both uncompressed and compressed powders. Powder XRD profiles were simulated using the Mercury software and crystallographic information files (CIFs) (see Fig. 3a–c). The CIFs of the pentacene, C60, and C8-BTBT single crystals were obtained from The Cambridge Crystallographic Data Centre (CCDC numbers are 619978 and 679293) and National Institute of Advanced Industrial Science and Technology^25^. The simulated XRD profiles are in good agreement with the experimental XRD profiles, indicating that crystal axes of each crystallite are random in the compressed powders. Additionally, the intensities of the diffraction peaks decreased and their full width at half maximum (FWHM) increased after CIP. The increasing rates of the FWHM are calculated to be about 18% from the (001) peaks of penacene, about 9% from the (111) peaks of C60, and about 22% from the (002) peaks of C8-BTBT. The intensity decrease and FWHM increase indicate that the crystallites are smashed into small pieces with the application of the 200-MPa pressure. The small pieces may fill the gaps between the powder particles, resulting in the smooth surface formed on the compressed powder as shown in Fig. 2b,c. The changes in the XRD profiles are similar in every compressed powder. It is known that a vertical orientation of molecules with their molecular axes stacked parallel to each other on a substrate surface enhances the carrier mobility in the stacking direction because of enhanced π coupling between neighboring molecules^26^,^27^. Therefore, the random crystal-axis directions in the compressed powders result in a low carrier mobility in the direction parallel to the substrate plane.

The XRD profiles of the vacuum-deposited films of pentacene, C60, and C8-BTBT were measured for comparison. No diffraction peaks were observed from the vacuum-deposited C60 film, indicating an amorphous-like film formation (Fig. 3b). However, the vacuum-deposited films of pentacene and C8-BTBT show the (00l) reflection peaks only (Fig. 3a,c). The peak positions in the vacuum-deposited pentacene film slightly shift to lower 2theta in comparison with the compressed pentacene powder because of formation of the pentacene thin film phase, not the single-crystalline bulk phase^28^. The calculated interlayer spacings from the (00l) peaks using the Bragg’s law are 15.7 Å for pentacene and 29.4 Å for C8-BTBT. These values correspond to the lengths of their long molecular axes^28^,^29^. This observation is proof of the vertical molecular orientations of pentacene and C8-BTBT on the substrates. Carrier transport parallel to the substrate is efficient in the vacuum-deposited films because of better π stacking in this direction.

### OFET characteristics of CIP-compressed powders

**Figure 4:**
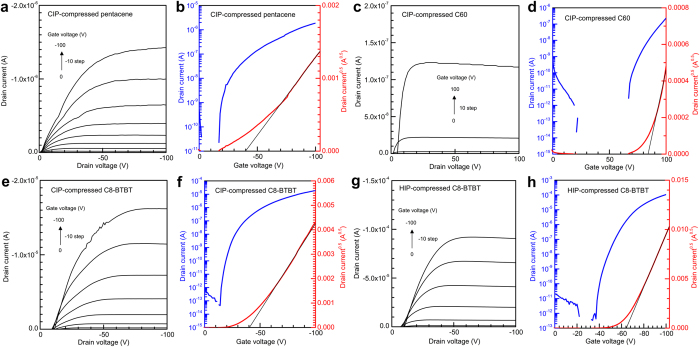
Representative output and transfer characteristics of OFETs fabricated with the CIP-compressed powders of (**a,b**) pentacene, (**c,d**) C60, and (**e,f**) C8-BTBT and (**g,h**) the HIP-compressed C8-BTBT powders. The black solid lines are the fitting results calculated using the saturation-regime metal-oxide semiconductor equation.

The uncompressed powders did not carry current. However, we were able to observe *p*-type FET characteristics from the CIP-compressed powders of pentacene and C8-BTBT and *n*-type FET characteristics from the compressed C60 powder because the gaps between the powder particles and the other particles, substrates, or electrodes were crushed using CIP. Representative output and transfer characteristics of the compressed powders are shown in Fig. 4. The drain currents did not begin to rise from 0 V in most OFETs, probably because the electrical contacts between the source-drain electrodes and the compressed powders are not enough. The carrier mobilities (*μ*) and threshold voltages (*V*_th_) were calculated from the plots of the square root of drain current versus gate voltage (*V*_g_) using a saturation-regime metal-oxide semiconductor equation^30^,


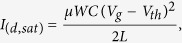


where *I*_d,sat_ is the saturated drain current, *W* is the channel width, *L* is the channel length, and *C* is the capacitance per unit area of the SiO_2_ insulator. The calculated carrier mobilities, threshold voltages, and current on/off ratios of the compressed powders and vacuum-deposited films are summarized in Table 1. The carrier mobilities of the vacuum-deposited films were comparable to those reported previously^28^,^31^,^32^.

The hole mobility in the saturation regime of the CIP-compressed pentacene powder was *μ*_h _= (1.2 ± 0.2) × 10^–3 ^cm^2^/V s (Fig. 4a,b and Table 1). This value is 1/78 of the hole mobility of the vacuum-deposited pentacene film [*μ*_h _= (9.4 ± 0.4) × 10^–2^ cm^2^/V s]. The reason for the lower hole mobility of the compressed powder is probably because there is not enough continuity between the powder particles and because the random crystal-axis directions of the crystallites are disadvantageous to hole transport.

C60 powder was also compressed using CIP. The electron mobilities (*μ*_e_) were *μ*_e _= (7.6 ± 5.4) × 10^−3^ cm^2^/V s for the compressed C60 powder and *μ*_e_ = (1.8 ± 0.9) × 10^−1^ cm^2^/V s for the vacuum-deposited C60 film (Fig. 4c,d and Table 1). The electron mobility of the compressed C60 powder is about 6 times higher than the hole mobility of the compressed pentacene powder. However, there is not a big difference in the carrier mobility between the vacuum-deposited films of pentacene and C60. C60 is spherically shaped molecules. Therefore, it is assumed that the randomness of the crystal-axis directions observed from the compressed C60 powder causes a small effect on the electron transport, resulting in the higher mobility.

C8-BTBT is known to be a material with a very high hole mobility over 1 cm^2^/V s 12,14,29,32. In fact, our vacuum-deposited C8-BTBT film had *μ*_h _= 3.9 ± 0.5 cm^2^/V s (Table 1). Therefore, we fabricated OFETs containing the CIP-compressed C8-BTBT powder. The hole mobility of the compressed C8-BTBT powder was *μ*_h _= (1.6 ± 0.4) × 10^–2^ cm^2^/V s (Fig. 4e,f and Table 1), which is the highest among the three materials investigated in this study.

Besides the carrier mobilities, the threshold voltages and current on/off ratios are also changed by the materials and fabrication processes (see Table 1). For example, the threshold voltages and current on/off ratios of the compressed powders of pentacene and C60 are lower than those of the vacuum-deposited films. However, this is not true in the C8-BTBT case. The cause of the changes of the threshold voltages and current on/off ratios is still unclear. Studies are currently in progress to understand these changes.

### HIP-compressed C8-BTBT powder

We performed HIP on C8-BTBT powder with the aim to further enhancing the carrier mobility. The HIP process is similar to the CIP process shown in Fig. 1, but the water temperature was kept at 90 °C under 200-MPa pressure. The hole mobility of the HIP-compressed powder reached *μ*_h _= 0.22 ± 0.07 cm^2^/V s (Fig. 4g,h and Table 1). This value is about one order of magnitude higher than that of the CIP-compressed C8-BTBT powder and comparable to that of amorphous silicon.

To investigate the reason for the mobility enhancement, the XRD profile of the HIP-compressed C8-BTBT powder was measured (Fig. 3d). In contrast to the XRD profiles of the CIP-compressed powders shown in Fig. 3c, the intensities of the diffraction peaks increased and their FWHM decreased by about 12% [the (002) plane] after HIP. This increase in the diffraction intensity and decrease in the FWHM indicate growth of the smaller crystallites to larger ones, resulting in the mobility enhancement. We note that the crystal-axis directions are still random in the HIP-compressed powder from the results of the XRD profiles.

The SEM images of the channel regions shown in Fig. 3e,f provide further confirmation that HIP enhances the hole mobility. Boundaries between the powder particles are observed in the CIP-compressed powder to some extent (Fig. 3e). The HIP-compressed powder has a more continuous surface with less particle boundaries (Fig. 3f). Although the water temperature during HIP is lower than the crystal-to-liquid crystal transition temperature (111.2 °C), the liquid crystal-to-isotropic liquid transition temperature (126.5 °C), and the melting temperature (129–131 °C) of C8-BTBT^29^, C8-BTBT powder may soften even at a mild temperature. The application of the high pressure to the soft powder probably leads to the improved continuity between the powder particles, resulting in the mobility enhancement.

## Discussion

The OFET characteristics of the compressed powders were markedly degraded in the presence of impurities and active gas such as oxygen in the powder (Fig. S1 and Table S1) because these species cause carrier scattering and carrier doping and work as carrier traps when directly incorporated into the compressed powders^33^,^34^,^35^. The OFET characteristics of the vacuum-deposited films were not sensitive to these species (Table S1) because the concentration of these species reduces during material evaporation. Therefore, every base powder used in this study was carefully purified using vacuum train sublimation and heated at 100 °C for 1 h in a nitrogen-filled glove box for degassing of the base powder just prior to CIP and HIP.

CIP and HIP were also tested on organic powders without the cover substrate, where the polymer bag was in contact with organic powder during CIP and HIP. The resulting compressed powders did not show typical OFET characteristics. This could be because smashing the powder particles into small pieces and just filling the gaps with the pieces is not sufficient to form continuous films because the polymer bag is too soft to smash the particles. The application of high pressure to the organic powder by placing it between two hard substrates is the key to induce smashing of the powder particles and filling the gaps effectively.

The phenyl termination of the SiO_2_ and Au surfaces is important to obtain better reproducibility of the OFET operation. Only 33% of the total number of the compressed powders on the unterminated SiO_2_ and Au surfaces showed OFET characteristics with large standard deviations. The efficiency of carrier injection from the untreated electrodes to the compressed powder may vary widely because of weak adhesion between them. The compressed powder often delaminated from the unterminated surface when the upper methyl-terminated substrate was removed from the compressed powder. The phenyl termination improves the adhesion and therefore increases the reproducibility up to 78% with a relatively small standard deviation as shown in Table 1.

**Figure 5:**
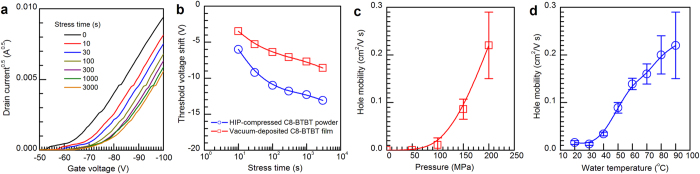
(**a**) Changes in the transfer characteristics before and after stressing the HIP-compressed C8-BTBT powders under constant bias voltages (source-drain voltage = gate voltage = –100 V) and (**b**) Plots of threshold voltage shifts vs stress time of the HIP-compressed C8-BTBT powders and vacuum-deposited C8-BTBT films. (**c**) Dependence of the applied pressure on the hole mobilities of the compressed C8-BTBT powders with a constant treatment temperature (90 °C) and a constant treatment time (60 min) and (**d**) dependence of the treatment temperature on the hole mobilities of the compressed C8-BTBT powders for a constant pressure (200 MPa) and a constant treatment time (60 min).

A bias stress test was performed on the OFETs containing the HIP-compressed C8-BTBT powders and vacuum-deposited C8-BTBT films. Figure 5a shows the changes in the transfer characteristics before and after stressing the HIP-compressed C8-BTBT powders under constant bias voltages (source-drain voltage = gate voltage = −100 V). The threshold voltages were found to shift to negative gate voltages as the stress time increased while *μ* was almost unchanged. Similar changes were observed in the vacuum-deposited C8-BTBT films. The threshold voltage shifts as a function of bias stress time are plotted in Fig. 5b. The threshold voltage shift is more significant in the HIP-compressed C8-BTBT powders than the vacuum-deposited C8-BTBT films. Although the source of the threshold voltage shifts is still unclear, it has been reported that charge carrier trapping in a dielectric or between grain boundaries induces threshold voltage shifts^36^,^37^. Therefore, we only speculate that holes traps are somehow easy to be formed in the HIP-compressed C8-BTBT powders under the constant bias condition.

The hole mobilities of the compressed C8-BTBT powders were found to increase with a strong dependence on the applied pressure and treatment temperature. The hole mobilities were zero up to 50 MPa and began to increase after 50 MPa as the applied pressure increased for a constant treatment temperature (90 °C) and a constant treatment time (60 min) (Fig. 5c). As the treatment temperature was increased with a constant pressure (200 MPa) and a constant treatment time (60 min), the hole mobilities increased (Fig. 5d). Therefore, we chose HIP compression conditions of 200 MPa and 90 °C for the powders. Although our equipment is limited to a maximum pressure of 200 MPa and a maximum water temperature of 90 °C, application of higher pressures to the powders at higher temperature is expected to result in a further enhancement of the hole mobilities and bias stability.

We introduced CIP to compress organic powder directly on a substrate. Using this method, we demonstrated operation of OFETs based on the compressed powder because the spatial gaps between powder particles and the other particles, substrates, or electrodes were crushed by application of 200-MPa pressure, resulting in *μ*_h _= 1.6 × 10^–2 ^cm^2^/V s from the CIP-compressed C8-BTBT powder. We also performed HIP using 90 °C hot water on C8-BTBT powder. The hole mobility of the HIP-compressed powder achieved *μ*_h _= 0.22 cm^2^/V s close to amorphous silicon because of growth of the C8-BTBT crystallites and a further improvement in the continuity between the powder particles. This powder compression using CIP and HIP means that a costly vacuum deposition system and harmful organic solvents are not necessary for the organic semiconductor layer fabrication, and therefore is applicable to manufacture low-cost OFETs in the future.

## Methods

### Substrate preparation

The *n*-type Si substrates with thermally grown SiO_2_ with a thickness of 300 nm were cleaned in a mixture of hydrogen peroxide and sulfuric acid and then rinsed with pure water several times. A 0.5-nm-thick Cr adhesion layer and a 50-nm-thick Au layer were vacuum deposited on top of the cleaned SiO_2_ surface and patterned by conventional photolithography, forming the Au source-drain electrodes with a channel length of 30 μm and a channel width of 2 mm. After the substrates were cleaned by ultrasonication in detergent, pure water, and isopropanol and finally ultraviolet-ozone treatment, the SiO_2_ and Au surfaces were treated with PTS and TP as reported in refs 38,39. The Si substrate used to cover the organic powder was cleaned in the same manner and then treated with HMDS by spin coating^28^.

### OFETs fabrication

Organic powder placed between the phenyl- and methyl-terminated substrates was vacuum sealed inside a thin flexible polymer bag (nylon-based multilayer sheets with a ~30-μm thickness). Then, CIP and HIP were performed on the vacuum-sealed samples as shown in Fig. 1. The water temperature was at room temperature (about 20 °C) for CIP and at 90 °C for HIP. At the present stage, it was difficult to control the amount of initial organic powder put on the substrate and the thickness of the compressed powder precisely. The typical thickness of our compressed powders was several hundred micrometers. OFETs were fabricated by vacuum deposition of a 50-nm-thick layer of pentacene, C60, or C8-BTBT on the phenyl-terminated substrates for comparison.

### Characterization of compressed powders and OFETs

The uncompressed and compressed powders were analyzed with SEM using a 5-kV accelerating voltage (JCM-5700, JOEM) and the XRD using a 2*θ*/*θ* technique [*λ* = 1.54 Å (CuKα)] (Ultima IV, Rigaku). The OFET characteristics of the compressed powders and vacuum-deposited films were measured with a semiconductor parameter analyzer (B1500A, Agilent Technologies) in vacuum at room temperature.

## Additional Information

**How to cite this article**: Matsushima, T. *et al.* Vacuum-and-solvent-free fabrication of organic semiconductor layers for field-effect transistors. *Sci. Rep.* **5**, 14547; doi: 10.1038/srep14547 (2015).

## Supplementary Material

Supplementary Information

## Figures and Tables

**Table 1:** Summary of *μ*, *V*_th_, and current on/off ratios of the compressed powders and vacuum-deposited films. The average values and standard deviations shown in this table were calculated for at least three different batches of the devices fabricated under identical fabrication conditions.

Sample	*μ* (cm^2^/V s)	*V*_th_ (V)	Current on/off ratio
CIP-compressed pentacene powder	(1.2 ± 0.2) × 10^–3^ (hole)	–41 ± 3	(9.2 ± 3.3) × 10^3^
Vacuum-deposited pentacene film	(9.4 ± 0.4) × 10^–2^ (hole)	–21 ± 2	(3.1 ± 1.1) × 10^5^
CIP-compressed C60 powder	(7.6 ± 5.4) × 10^–3^ (electron)	83 ± 2	(8.2 ± 17) × 10^4^
Vacuum-deposited C60 film	(1.8 ± 0.9) × 10^–1^ (electron)	68 ± 9	(1.5 ± 3.0) × 10^7^
CIP-compressed C8-BTBT powder	(1.6 ± 0.4) × 10^–2^ (hole)	–39 ± 7	(1.3 ± 1.8) × 10^8^
Vacuum-deposited C8-BTBT film	3.9 ± 0.5 (hole)	–68 ± 2	(7.6 ± 12) × 10^6^
HIP-compressed C8-BTBT powder	0.22 ± 0.07 (hole)	–65 ± 3	(4.2 ± 4.5) × 10^8^
