# Supplementary material for: Vacuum-and-solvent-free fabrication of organic semiconductor layers for field-effect transistors
Source: Sci Rep. 2015 Sep 29;5:14547. doi: 10.1038/srep14547 (PMC4586892; doi:10.1038/srep14547)
Supplement: Supplementary Information [file srep14547-s1.doc]

**Supplementary Information**

**Vacuum-and-solvent-free fabrication of organic semiconductor layers for field-effect transistors**

Toshinori Matsushima^1,2,*^, Atula S. D. Sandanayaka^1,2^, Yu Esaki^1^,

and Chihaya Adachi^1,2,*^

^1^Center for Organic Photonics and Electronics Research, Kyushu University, 744 Motooka, Nishi, Fukuoka 819-0395, Japan. ^2^Japan Science and Technology Agency (JST), ERATO, Adachi Molecular Exciton Engineering Project, 744 Motooka, Nishi, Fukuoka 819-0395, Japan. Correspondence and requests for materials should be addressed to C.A. (email: adachi@cstf.kyushu-u.ac.jp) or to T.M. (email: tmatusim@opera.kyushu-u.ac.jp).

**Influence of impurities and oxygen on OFET characteristics of compressed powders.**

The OFET characteristics of the compressed powders were markedly degraded in the presence of impurities and active gas such as oxygen in the powder. Figure S1 shows the representative output and transfer characteristics of OFETs fabricated with the CIP-compressed powders that were made from as-purchased pentacene powder. The calculated *μ*, *V*_th_, and current on/off ratios are summarized in Table S1. Although *V*_th_ of *p*-type OFETs should be a negative value, the actual *V*_th_ of the OFETs was found to be a positive value (54±7 V). Additionally, the *μ* was low [(3.7±1.7)×10^–4^ cm^2^/V s] and the current on/off ratio was very small (3±1) for the OFETs. The positive *V*_th_ and very small on/off ratio are because of a very high off-current (Figs. S1a and S1b). As-purchased pentacene powder is expected to include impurities and oxygen, resulting in the degraded OFET characteristics because these species cause carrier scattering and carrier doping and work as carrier traps when directly incorporated into the compressed powders.

As-purchased pentacene powder was carefully purified using vacuum train sublimation to remove the impurities. Use of the purified powder led to an enhancement of *μ*, *V*_th_, and current on/off ratio (Figs. S1c and S1d and Table S1). However, the *V*_th_ was still positive (9±3 V) and the on/off ratio was low (38±4) with the *μ* increasing about 2.6 times to (9.5±2.7)×10^–4^ cm^2^/V s. As the purified powder was taken out in air after the sublimation, the purified powder might absorbs oxygen again. Oxygen absorbed in the purified powder is assumed to be the source of the positive *V*_th_ and small on/off ratio. On the other hand, it was confirmed that the OFET characteristics of the vacuum-deposited films were not sensitive to these species (Table S1) because the concentration of these species reduces during material evaporation.

The purified powder was heated at 100 °C for 1 h in a nitrogen-filled glove box for degassing of the purified powder just prior to CIP and HIP. Degassing using this method was very effective for improving the OFET characteristics (Figs. S1e and S1f and Table S1), especially for the *V*_th_ (–41±3 V) and current on/off ratio [(9.2±3.3)×10^3^] while the *μ* was not changed significantly [(1.2±0.2)×10^–3^ cm^2^/V s]. **Therefore, every base powder used in this study was purified and heated in the same manners.**

**
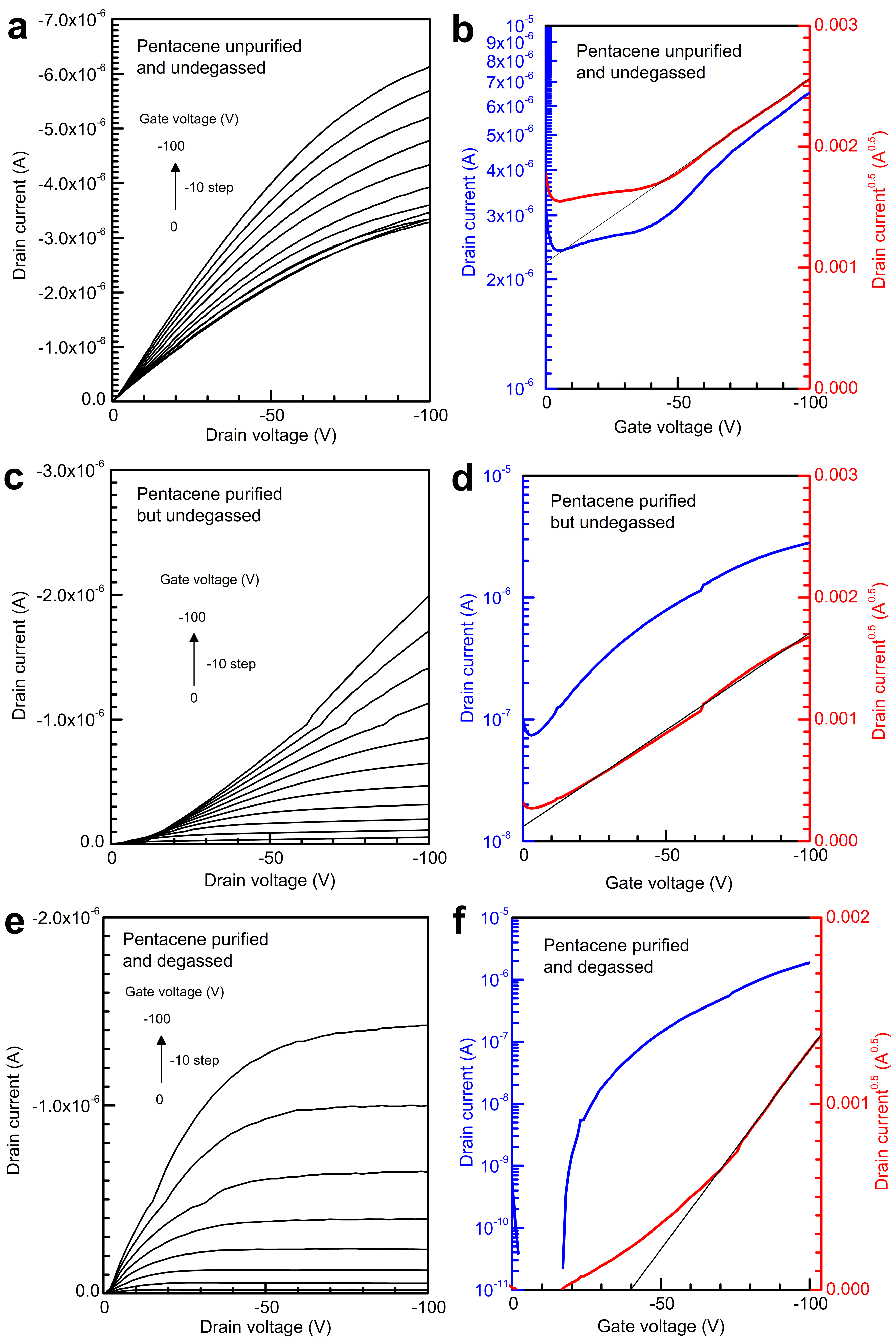
**

**Figure S1.** Representative output and transfer characteristics of OFETs fabricated with the CIP-compressed pentacene powders. (**a,b**) Pentacene powder unpurified and undegassed, (**c,d**) pentacene powder purified but undegassed, and (**e,f**) pentacene powder purified and degassed. The black solid lines are the fitting results calculated using the saturation-regime metal-oxide semiconductor equation. (**e,f**) are the same as Figs. 4a and 4b.

**Table S1.** Summary of *μ*, *V*_th_, and current on/off ratios of the CIP-compressed pentacene powders and vacuum-deposited pentacene films. The fifth row of this table is the same as the second row of Table 1.

| Sample | *μ* (cm^2^/V s) | *V*_th_ (V) | Current on/off ratio |
| --- | --- | --- | --- |
| CIP-compressed powder  (pentacene unpurified and undegassed) | (3.7±1.7)×10^–4^ | 54±7 | 3±1 |
| CIP-compressed powder  (pentacene purified but undegassed) | (9.5±2.7)×10^–4^ | 9±3 | 38±4 |
| CIP-compressed powder  (pentacene purified and degassed) | (1.2±0.2)×10^–3^ | –41±3 | (9.2±3.3)×10^3^ |
| Vacuum**-**deposited film  (pentacene unpurified) | (5.3±0.3)×10^–2^ | –27±3 | (2.6±1.5)×10^5^ |
| Vacuum**-**deposited film  (pentacene purified) | (9.4±0.4)×10^–2^ | –21±2 | (3.1±1.1)×10^5^ |
